# Sortilin in Glucose Homeostasis: From Accessory Protein to Key Player?

**DOI:** 10.3389/fphar.2018.01561

**Published:** 2019-01-15

**Authors:** Nicolas Blondeau, Sophie Béraud-Dufour, Patricia Lebrun, Céline Hivelin, Thierry Coppola

**Affiliations:** Centre National de la Recherche Scientifique, Institut de Pharmacologie Moléculaire et Cellulaire, UMR 7275, Université Côte d’Azur, Valbonne, France

**Keywords:** diabetes, receptor, pharmacology, signaling, physiology

## Abstract

The pharmacological properties and physiological roles of the type I receptor sortilin, also called neurotensin receptor-3, are various and complex. Sortilin is involved in important biological functions from neurotensin and pro-Nerve Growth Factor signaling in the central nervous system to regulation of glucose and lipid homeostasis in the periphery. The peripheral functions of sortilin being less extensively addressed, the focus of the current review is to discuss recent works describing sortilin-induced molecular mechanisms regulating blood glucose homeostasis and insulin signaling. Thus, an overview of several roles ascribed to sortilin in diabetes and other metabolic diseases are presented. Investigations on crucial cellular pathways involved in the protective effect of sortilin receptor on beta cells, including recent discoveries about regulation of cell fate, are also detailed. In addition, we provide a special focus on insulin secretion regulation involving complexes between sortilin and neurotensin receptors. The last section comments on the future research areas which should be developed to address the function of new effectors of the sortilin system in the endocrine apparatus.

## Sortilin Receptor

The protein sortilin, composed of 833 amino acids in human, is a high affinity receptor (Kd ≈ 0.3 nM) for NT (Mazella et al., 1989, 1998). It was first identified in 1997 as a receptor-associated protein (RAP) (Petersen et al., 1997). Sortilin belongs to the Vps10p family of type I receptors, with SorLA and SorCS1-3 receptors (Jacobsen et al., 1996; Hermey et al., 1999). The members of this family are characterized by a single transmembrane domain framed by an extracellular domain rich in cysteines (comparable to that of the Vps10p trivalent vacuolar protein of yeast), and a short intracellular end (C-terminal domain) involved in its internalization (Petersen et al., 1996, 1997; Mazella et al., 1998). Sortilin is widely expressed in the central nervous system, particularly in the hippocampus, dentate gyrus and cerebral cortex (Hermans-Borgmeyer et al., 1999). It is also present in the spinal cord, skeletal muscle, testes, heart, placenta, pancreas, prostate, and small intestine (Petersen et al., 1997). Sortilin distribution in the cell is highly regulated. While it is very poorly present at the PM (5–10%), the bulk of the protein localizes in intracellular compartments (vesicles and trans-golgi network) (Morris et al., 1998). If three NT receptors (NTSRs) NTSR1, NTSR2 and NTSR3 (or sortilin) mediate the effects of NT, the NTSR1 and NTSR2 receptors belong to the large GPCR (G Protein-Coupled Receptor) family in contrast to sortilin that is a sorting receptor with a single transmembrane domain (Mazella et al., 1989, 1998).

Sortilin is synthesized in an immature form, prosortilin. Prosortilin convertion to its mature receptor occurs in the Golgi apparatus through removal of its N terminal domain by the pro-convertase furin. This leads to the release of a peptide of 44 amino acids (SDP for sortilin derived propeptide: Gln1-Arg44, also known as PE). While such maturation process is a general mechanism to control receptor or enzyme activation, in the present case, the canonical furin cleavage leads also to the release of a new active peptide. Indeed, SDP binds to mature sortilin and TREK-1 channel with a high affinity (Kd_∼_5nM) (Munck Petersen et al., 1999; Mazella et al., 2010). Several groups have also largely documented that sortilin is a co-receptor of the prodomain of proNGF and proBDNF (Nykjaer et al., 2004; Teng et al., 2005; Arnett et al., 2007).

**FIGURE 1:**
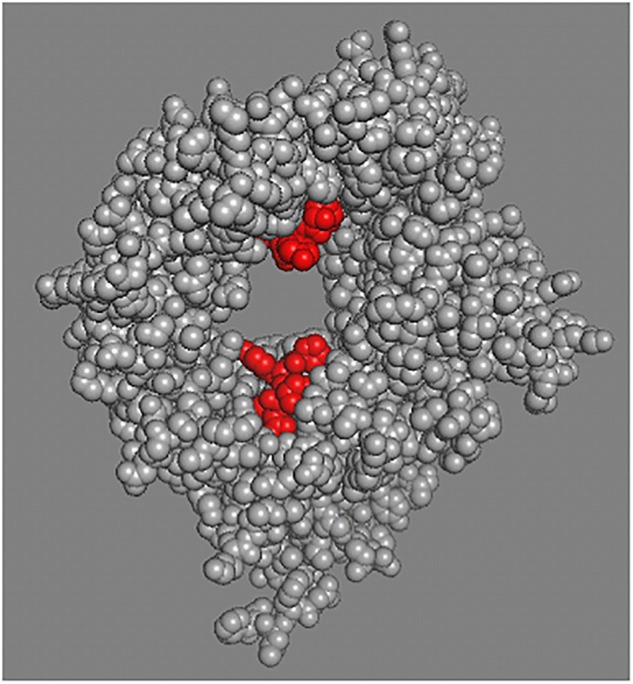
Sortilin structural model. Sortilin complexed with NT structural model PDB 3F6K. The extracellular domain of sortilin, receptor for NT was crystallized and the tridimensional structure at 2A resolution was determined. Peptides binding (two sites) relates to the restricted space inside the tunnel of the b-propeller (Quistgaard et al., 2009, 2014). Sortilin is shown in gray and NT in red.

Even though the binding properties of each sortilin ligand have not been clearly unraveled, data suggest that binding sites are similar for most of them (Quistgaard et al., 2009). As illustrated by Figure 1, NT (in red) binds to sortilin (in gray) in a tunnel of a ten-bladed β-propeller domain. The structural properties of sortilin suggests that NT is completely contained inside the tunnel of the β-propeller, firmly bound by its C-terminus and further attached by its N-terminal part at a low affinity site on the opposite side of the tunnel (PDB: 3F6K).

## Sortilin is Associated to Distincts NTSRs to Modulate NT-Mediated Endocrine Cell Functions

Physiological studies performed during the first years following the discovery of NT in 1973, revealed some interesting results concerning the potential involvement of the peptide in glucose homeostasis and lipid absorption. Indeed in human, NT is released in the blood circulation after a meal, and lipid absorption stimulates NT release from the rat small intestine (Leeman and Carraway, 1982). In addition, NT is released from pancreas in STZ-diabetic rats (Berelowitz and Frohman, 1982) and is co-localized with glucagon in the endocrine human fetal pancreas (Portela-Gomes et al., 1999). Interestingly, NT exerts a dual effect on the rat endocrine pancreas: at low glucose concentration, the peptide stimulates insulin and glucagon release whereas at high glucose concentration, it has the opposite effect (Dolais-Kitabgi et al., 1979). Furthermore, NT administration increases pancreatic weight and DNA content indicating a prominent proliferative effect of the peptide on pancreatic cells (Feurle et al., 1987; Wood et al., 1988).

**FIGURE 2:**
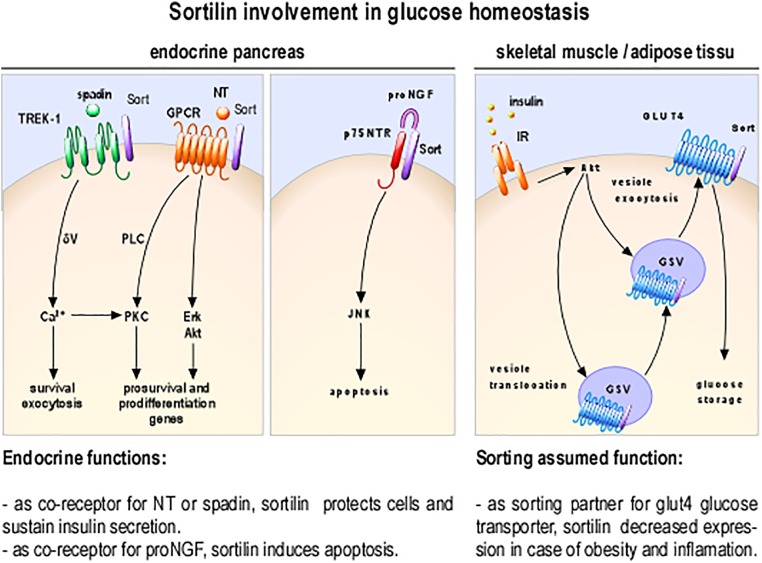
Cellular and molecular processes involving sortilin as receptor or co-receptor. Sortilin (in purple) as NT regulation via the complex of GPCR/sortilin; the complex p75NTR/sortilin promotes apoptosis inducing JNK dependent pathway, spadin inhibition of TREK-1 K+ currents potentiate insulin secretion and as sorting partner is a part of the machinery necessary for insulin dependent translocation of GLUT4 storage vesicles (GSV); insulin receptor (IR).

As involvement of NT in glucose homeostasis become clearer, understanding the mechanisms of its action on insulin-secreting cells is crucial, especially because RT-PCR and Western blot analyses have demonstrated that all NTSRs are expressed in rat and mouse islets and in insulin-secreting beta cell lines (Coppola et al., 2008; Beraud-Dufour et al., 2009). Functionally, NT can modulate various biological responses both in insulinoma derived cell lines and in isolated pancreatic islets (Khan et al., 2017; Figure 2) suggesting the possibility of differential activation pathways by NT. In this area, some studies indicated that the PKC proteins play a key role in the direct effect of NT on insulin secretion from beta cells (Beraud-Dufour et al., 2010; Khan et al., 2017).

Clinical data, by showing that circulating NT levels are increased in human diabetes and obesity (Melander et al., 2012; Chowdhury et al., 2016) identified NT regulation as a promising therapeutic target in these most prevalent and challenging health conditions. This was correlated by *in vivo* studies showing that NT (NT-/-) and sortilin KO mice (Sort1-/-) share some common phenotypes, especially by protecting from obesity, hepatic steatosis, and metabolic disorders. Indeed, sortilin deficiency induces a beneficial metabolic phenotype in liver and adipose tissue against high fat diet (Rabinowich et al., 2015).

Furthermore, studies on double knockout mice for the low-density lipoprotein receptor (Ldlr-/-, an atherosclerosis model) and sortilin (Sort1-/-) have confirmed the previous observations showing that sortilin is crucial for lipid homeostasis by suppressing intestinal cholesterol absorption mostly in female mice (Hagita et al., 2018). It is important to note that some contradictory results were obtained using another so-called sort1-/- model (Li et al., 2017a). It was observed that suppression of sortilin gene does not affect diet-induced obesity and glucose uptake from adipose tissue and skeletal muscle. A closer look of both Sort1-/- model mice used showed that the first studies were performed using a mouse in which the exon 14th of the sortilin gene was deleted, leading to the expression of a soluble sortilin receptor (Rabinowich et al., 2015; Hagita et al., 2018).

The other deletion was done on the second exon deleting almost all of the sortilin protein (Li et al., 2017a). Similarly to the first Sort1-/- model described (Rabinowich et al., 2015; Hagita et al., 2018), NT-deficient mice are resistant to DIO, hepatic steatosis and insulin resistance (Li et al., 2016). In order to explain such differences between sortilin deficient mice models, it is possible to argue that the expression of a circulating soluble form of the receptor, is able to buffer the NT in the blood stream and also potentially the other sortilin-interacting peptides (Quistgaard et al., 2014). Thus, we can postulate that the truncated receptor depletes circulating NT, similarly to a NT KO- phenotype. In addition, the metabolic improved phenotype (protection from obesity, hepatic steatosis, and insulin resistance) observed in NT-deficiency suggests an involvement of sortilin in NT-regulation of glucose homeostasis.

In correlation with this postulate, it has been shown that NT has a potent anti-apoptotic effect against IL-1bβ or staurosporine (Coppola et al., 2008). This protective effect of NT is mediated by sortilin in combination with NTSR2 (Beraud-Dufour et al., 2009). Sortilin as a co-receptor with NTSR2 is necessary for the anti-apoptotic NT function and also for the peptide modulation of insulin secretion (Beraud-Dufour et al., 2010). This receptor heterodimer exerts a protective effect against apoptosis by stimulating PI-3 kinase activity which in turn phosphorylates Akt (Coppola et al., 2008). Importantly, recent works performed in rodent and human beta cells, showed that NT was also produced inside the islets and regulates adaptation to environmental stress (Khan et al., 2017).

These results, all together, underline that sortilin, as receptor or co-receptor for NT, may have a dual and beneficial role: protecting endocrine cells from stress induced apoptosis and/or controlling lipid absorption from the intestine.

## Sortilin, a Sorting Protein for Glucose Transporter GLUT4, is a Key Modulator of Metabolism

As largely documented, sortilin and the glucose transporter GLUT4 are co-expressed in differentiated adipocytes and myotubes (Figure 1; Bogan and Kandror, 2010), and are necessary for glucose storage. Fine-tuning of the expression level of both proteins is crucial in order to maintain insulin mediated glucose transport inside the cells (Figure 2). For example, formation of Glut4 storage vesicles in adipocytes and skeletal muscle cells correlates with the expression of sortilin (Ariga et al., 2008; Shi et al., 2008; Ariga et al., 2017). In addition, insulin dependent translocation of Glut4 is clearly correlated with the presence of sortilin in adipocytes (Huang et al., 2013). It has been hypothesized that defect in peripheral glucose transport, related to insulin resistance observed in obesity and diabetes, could be correlated *in vivo*, with modification of the expression level of sortilin (Kaddai et al., 2009).

In addition, sortilin expression is decreased in several physiopathological conditions such as obesity and this repression is TNF-alpha dependent. Interestingly, a link between chronic low-grade inflammation, sortilin expression and insulin resistance has been postulated (Kaddai et al., 2009). Indeed, sortilin expression is differently altered in insulin resistance models induced by TNF-alpha or dexamethasone treatments. In presence of TNF-alpha the expression of sortilin is drastically decreased and can be associated with insulin resistance; on the opposite dexamethasone dependent insulin resistance is not accompanied by sortilin downregulation (Hivelin et al., 2017; Li et al., 2017b). TNF-alpha combined with hypoxia was most able to mimic *in vivo* DIO-induced adipose insulin resistance (Lo et al., 2013). Also, sortilin gene expression is decreased in white adipose tissue of obese mice when PI3K/AKT signaling is inhibited (Li et al., 2017b).

TNF-alpha and dexamethasone are inducers of insulin resistance, respectively inhibiting insulin receptor tyrosine kinase activity (Hotamisligil et al., 1996; Rui et al., 2001) or inducing whole body insulin resistance without affecting GLUT4 translocation machinery (Qi et al., 2004). All together these results show that down regulation of sortilin expression may be one of the events leading to insulin resistance.

## Sortilin, Acting on TREK-1 Channel, Regulates Cell Excitability

With respect to insulin resistance and secretion, potassium permeability by controlling the beta cell membrane potential regulates insulin secretion. Indeed, insulin granule exocytosis is induced by membrane depolarization that is a direct consequence of the inhibition of ATP dependent potassium channels by the increased ATP production from glucose metabolism. Other types of K+ channels including TREK-1, a two-pore-domain (K_2_P) background potassium channel are known to be involved in the control of the resting membrane potential and the regulation of depolarizing stimulus (Kim and Kang, 2015).

This is part of the K_2_P(s) general properties. Background K+ outward currents could adjust the membrane potential, low hyperpolarization or depolarization (Kim, 2005). Therefore, interfering with TREK-1 plasma conductance could play an important role in the electrophysiology of insulin secretion. TREK-1 channel could be thereby, a potential target for pharmacological agents designed to modulate this secretion. This has to be underlined, as we have recently reported that the TREK-1 channel is inhibited by SDP (propeptide: Gln1-Arg44), as well as by its shorter analog spadin (Mazella et al., 2010).

Since structure function studies determined that SDP region Gln17-Arg28 was as efficient in binding affinity (Kd_∼_5nM) than the entire peptide, we generated a TREK-1 inhibitory peptide called spadin (Sortilin Peptide AntiDepressive in) which has amino acids 17–28 preceded by sequence 12–16 (APLRP) to maintain conformational stress (Mazella et al., 2010). Results clearly demonstrated the capacity of spadin and SDP for blocking TREK-1 currents (Mazella et al., 2010). The bioactivity of SDP is of interest as a specific SDP dosing assay revealed that its circulating level is of significant concentrations (10 nM) in the mouse serum (Mazella et al., 2010). In addition, TREK-1 and sortilin are co-expressed in pancreatic islets, only in aα and β cells. At a cellular level TREK-1 and sortilin co-localize in intracellular compartments (Hivelin et al., 2016). Therefore we explored the inhibitory effects of spadin on endogenous TREK-1 current in β pancreatic cells (Hivelin et al., 2016). We could observe changes in resting membrane potential in MIN6B1 pancreatic beta cell line incubated in the presence of 10nM spadin. The mild depolarization observed (delta = 12.84 mV), although not sufficient to induce insulin secretion by itself, potentiates the effect of secretagogues such as 16.7 mM glucose or 30 mM KCl (Hivelin et al., 2016). This PM depolarization likely facilitates the exocytosis process through the enhancement of intracellular calcium concentration. Moreover, we reported that during *in vivo* IPGTT experiments, glucose level is always lower in mice treated with spadin, suggesting a direct action of spadin on glycaemia (Hivelin et al., 2016).

Interestingly most of the consequences of spadin inhibitory action on TREK-1 channel were primarily documented in neurons, and among them some interesting features could be relevant for beta cell mass retention. Spadin as specific inhibitor for TREK-1 channel currents induces survival pathways, such as Akt and Erk pathways in primary cultured neurons (Devader et al., 2015). Accordingly, this was associated with an anti-apoptotic effect, associated with an increase of the phosphorylation of CREB. *In vivo*, spadin induces a rapid onset of neurogenesis (Mazella et al., 2010). Therefore, it is tempting to postulate that such protective and modulation of the proliferation pathways (Abdelli et al., 2009; Beeler et al., 2009), could be observed in endocrine pancreatic cells that shear common traits with neurons. These traits are required for insulin secretion (Abderrahmani et al., 2004a,b).

## Sortilin and proNGF Partners in Death Fate: Sortilin Dependent Apoptosis

Since several years, a link between NGF and diabetes has been showed. For example, the NGF/proNGF expression ratio is decreased in the brain of diabetic rats (Soligo et al., 2015) and in STZ-induced diabetic rats (Sposato et al., 2007). Furthermore, these rats present an up-regulation of the p75NTR expression in the pancreas (Sposato et al., 2007), suggesting a role of this receptor in the higher apoptosis rate observed in the endocrine pancreas of these animals. The role of the sortilin/p75NTR receptor complex in the induction of death described in neurons (Nykjaer et al., 2004) has also been identified in lymphocytes (Rogers et al., 2010). It has been demonstrated that proNGF, when TrkA expression decreases, switches PC12 cells from growth to apoptosis (Ioannou and Fahnestock, 2017). Interestingly, NGF receptors (p75NTR and TrkA) are expressed in human and rodent pancreatic islets (Miralles et al., 1998; Rosenbaum et al., 1998; Pingitore et al., 2016) and NGF is expressed and secreted by adult beta cells, suggesting an autocrine effect (Rosenbaum et al., 1998).Thus, it is tempting to postulate that a decreased maturation of proNGF, as observed in the brain of diabetic rat (Soligo et al., 2015), could represent a major cause of beta cell apoptosis. In that perspective, it would be of great importance to carefully analyze the role of sortilin, TrkA, p75NTR, and NGF as pro- or anti-apoptotic promoters in beta cells.

## Conclusion/Perspectives

The reviewing of the literature undoubtedly identifies the high affinity NT receptor, sortilin, as involved in key regulatory mechanisms of glucose homeostasis. Although it is generally accepted that the results obtained on cells from Langerhans islets are preferable to any other, most of the data showing a specific role of sortilin in beta cell comes from tumor-derived cell lines Nevertheless, work on tumor lines, that retain much of the properties of differentiated cells (Merglen et al., 2004) is useful in understanding the role of sortilin. We cannot, at this stage, give a complete and perfectly defined role for sortilin in beta cell. However, it is possible to say, with caution, that sortilin could play a role in survival and function maintenance of beta cell, when associated with an NT receptor, or pro-apoptotic role when associated with p75NTR (Figure 2). These seemingly contradictory functions are not informed so far by *in vivo* studies emphasizing the usefulness of work on the endocrine pancreas of sort-/- mice.

In addition to the various drugs interfering with insulin secretion, design of new drugs targeting sortilin should be considered. Antagonism against proneurotrophin action, for example, could represent a possible pharmacological approach for beta cell mass preservation, which is regarded as crucial in the prevention and treatment of type 2 diabetes. Demonstration of the implication of K+ outward currents as target for insulin exocytosis potentiation by spadin is crucial for new pharmacological perspectives. However, the “druggability” of the sortilin-underlying pathways is complicated because of multiplicity of sortilin intricated functions and its large-scale inhibition could have deleterious effects on general metabolism. Further research is then required to provide evidence of the effectiveness and feasibility of sortilin pathways targeting for therapeutic intervention in obesity and type 2 diabetes.

## Author Contributions

NB and SB-D shared first co-authorship. All authors participated in writing the article.

## Conflict of Interest Statement

The authors declare that the research was conducted in the absence of any commercial or financial relationships that could be construed as a potential conflict of interest.

## Abbreviations

AKT: RAC-alpha serine/threonine-protein kinase
BDNF: brain derived neurotrophic factor
DIO: diet induced obesity
ERK: extracellular signal-regulated kinases
GPCR: G protein coupled receptor
IPGTT: intra peritoneal glucose tolerance test
K_2_P: two-pore-domain background potassium channel
Ldlr: low-density lipoprotein receptor
NGF: nerve growth factor
NT: neurotensin
NTSR2: neurotensin receptor-2
NTSR3: neurotensin receptor-3
p75NTR: neurotrophin receptor of 75 kDa
PKC: protein kinase C
PM: plasma membrane
STZ: streptozotocin
